# Factors influencing nasal airway pressure and comfort in high-flow nasal cannula oxygen therapy: a volunteer study

**DOI:** 10.1186/s12890-023-02752-6

**Published:** 2023-11-20

**Authors:** Enqi Zhao, Yilong Zhou, Chunwei He, Dedong Ma

**Affiliations:** 1 Qilu Hospital of Shandong University, Jinan, 250012 Shandong China; 2 Department of Respiratory and Critical Care Medicine, Qilu Hospital of Shandong University, Jinan, 250012 People’s Republic of China

**Keywords:** End-expiratory pressure, Comfort, Influencing factor, High-flow nasal cannula

## Abstract

**Background:**

High-flow nasal cannula (HFNC) oxygen therapy is essentially a constant-flow, noninvasive respiratory support system similar to a noninvasive ventilator operating in constant-flow mode. The clinical outcome of HFNC oxygen therapy is strongly associated with the pressure generated by high-flow gas and the patient’s comfort level. This study was performed to explore the relevant factors affecting pressure and comfort of HFNC oxygen therapy in vivo.

**Methods:**

Thirty-five healthy volunteers were enrolled in the trial. They underwent placement of nasal cannulas of various inner diameters (3, 4 or 5 mm) and treatment with different HFNC devices [HFT-300 (Weishengkang Medical Technology Co., Ltd., Jiangsu China) or H-80 M (BMC Medical Co., Ltd., Beijing China)],and the nasal airway pressure and comfort were assessed. Multiple linear regression was used to determine predictors of airway pressure.

**Results:**

Multiple linear regression showed that the end-expiratory pressure was associated with the flow rate, sex, height, and cannula size. The end-expiratory pressure increased by 0.6 cmH_2_O per 1-mm increase in cannula diameter, decreased by 0.3 cmH_2_O per 10-cm increase in participant height (with a 0.35 cmH_2_O decrease for men), and increased by 1 cmH_2_O when the flow rate increased by 10 L/min (R^2^ = 0.75, *P* < 0.05 for all variables in model). In addition, the pressure generated by the H-80 M device was higher than that generated by the HFT-300 device (*P* < 0.05). Discomfort manifested as difficulty in expiration, and its severity increased as the cannula diameter increased; however there was no significant difference in comfort between the two HFNC devices (*P* > 0.05).

**Conclusion:**

In volunteers undergoing HFNC oxygen therapy, the nasal cannula diameter, flow rate, sex, height, and device model can affect the nasal airway pressure, and the nasal catheter diameter and flow rate can affect comfort. These factors should be given close attention in clinical practice.

**Trial registration:**

ChiCTR2300068313 (date of first registration: 14 February 2023, https://www.chictr.org.cn).

**Supplementary Information:**

The online version contains supplementary material available at 10.1186/s12890-023-02752-6.

## Background

High-flow nasal cannula (HFNC) oxygen therapy is a new noninvasive respiratory support method that is being increasingly utilized in clinical practice. HFNC oxygen therapy provides heated and humidified oxygen at adjustable concentrations and flow rates through the nasal route. It is essentially a constant-flow, noninvasive support system that allows air leakage and is similar to a noninvasive ventilator operating in constant-flow mode. HFNC oxygen therapy is utilized in a variety of conditions, such as hypoxemic respiratory failure and obstructive sleep apnea. Notably HFNC oxygen therapy can improve clinical outcomes through a variety of mechanisms, including improvement in oxygenation [1], generation of positive intra-airway pressure [2–4], and reduction of airway resistance and respiratory work [5, 6]. The end-expiratory pressure (EEP) generated by this therapy can also reduce the pressure difference between the airway and alveoli, prevent airway closure and gas trapping, and to some extent, counteract endogenous positive end expiratory pressure [7, 8]. Previous studies have preliminarily evaluated the effect of nasal cannulas on airway pressure in vitro [9, 10]. In addition, studies have shown that the comfort and tolerance levels are higher with HFNC oxygen therapy than with nasal cannula oxygenation and noninvasive ventilation [11, 12]. Treatment intolerance is associated with failure of noninvasive respiratory after extubation of patients with chronic obstructive pulmonary disease [12], suggesting that comfort is associated with improved clinical outcomes. Both the level of positive pressure and the degree of comfort have a critical impact on the therapeutic effect. Both single-level and double-levels noninvasive ventilators enable direct monitoring and control of pressure. For HFNC oxygen therapy, the flow rate is the main regulatory parameter, and direct regulation of pressure is not possible. Therefore, the present study was performed to further explore the factors affecting pressure and comfort in HFNC oxygen therapy in vivo.

## Methods

All participants involved in this study gave their informed consent. The institutional review board of our hospital approved the study (No.KYLL-202210-24, November 2022). In total, 35 healthy adults (16 men, 19 women) aged 18 to 40 years were enrolled in this study, and all were university students. General demographic data were collected from all volunteers, including sex, age, height, weight, and body mass index (BMI). Individuals who had a history of upper respiratory infection in the past 2 weeks, had a history of smoking, or used drugs influencing cardiopulmonary function were excluded. Three different sizes (3, 4 and 5 mm) of nasal cannulas (Excellentcare Medical Ltd., Huizhou, Guangdong Province, China), which were typical of the most commonly used sizes in our hospital, and two different HFNC devices [HFT-300 (Weishengkang Medical Technology Co, Ltd., Jiangsu China) and H-80 M (BMC Medical Co, Ltd., Beijing China)] were used in the study.

### Test procedure

**Fig. 1:**
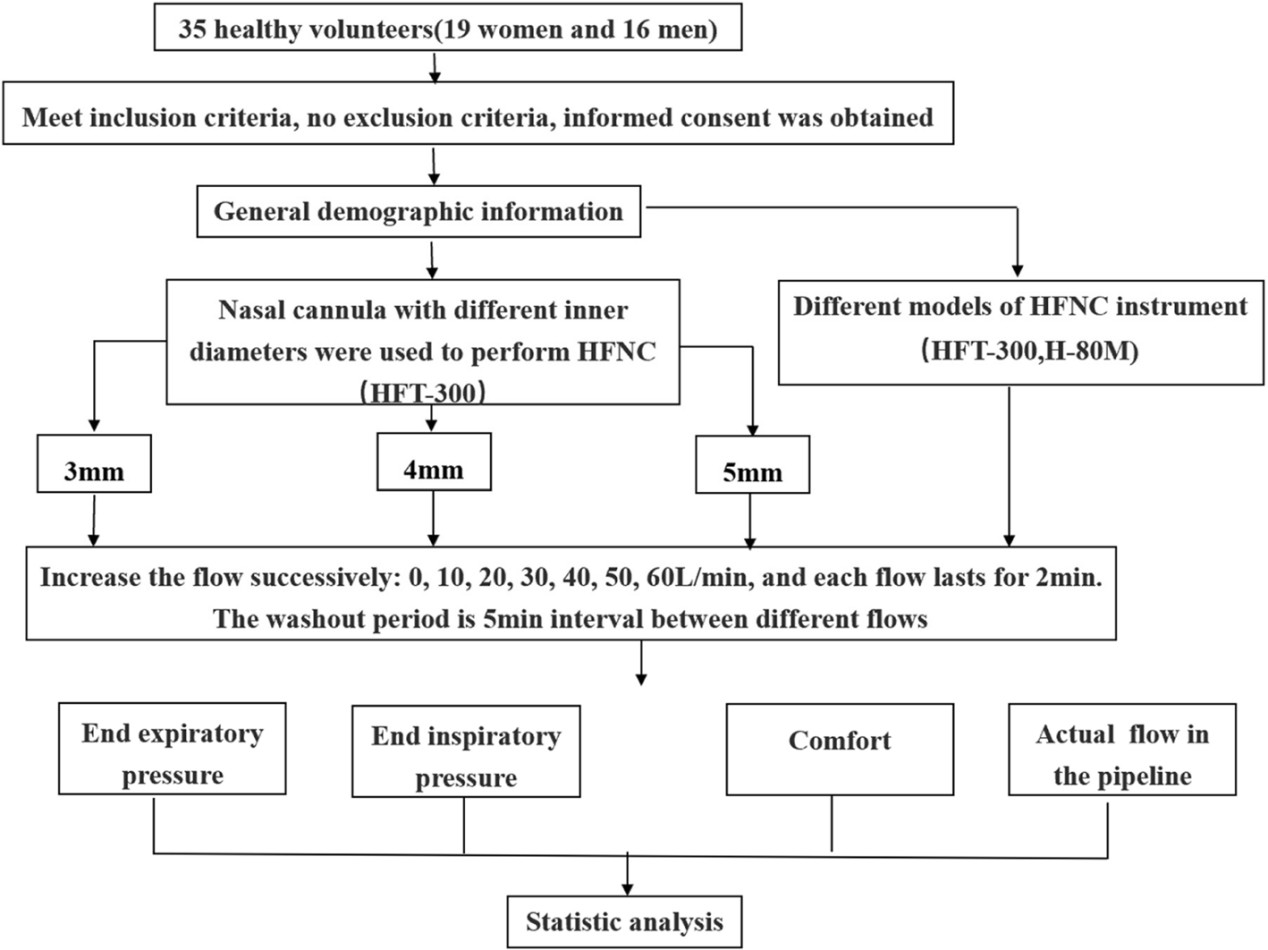
Flowchart

Before the test, all participants were given an explanation of the test procedure to ensure their understanding. The participants rested for 10 minutes before the start of the experiment to achieve a physiological steady state. During the study, the participant calmly sat in a vertical position with their mouth closed breathing through their nose (oxygen concentration of inhaled gas 21%, temperature 36 °C, and relative humidity 100%). A handheld digital manometer (AZ8252; AZ Instrument Corp., Taiwan, China) was used to measure the EEP and end-inspiratory pressure (EIP). One end of an anesthetic catheter (Henan Tuoren Medical Device Co., Ltd., Xinxiang, Henan, China) was inserted into the nasal cavity to a depth of 4 cm, and the other end was connected to the handheld digital manometer through the adapter. A previous study showed that the pressure does not increase at a nasal depth beyond 3 cm [13]. The manometer transmitted the pressure data to the computer in real time through software (Handheld Meter Data Logger version 3.10; Kingst History Data Review Beijing, China). The mean EEP and EIP were calculated by averaging the pressure from the peak of expiration and inspiration of each breath during the 2-minute recording (Fig. 1).

### Assessment of differences in pressure and comfort using different nasal cannulas

**Fig. 2:**
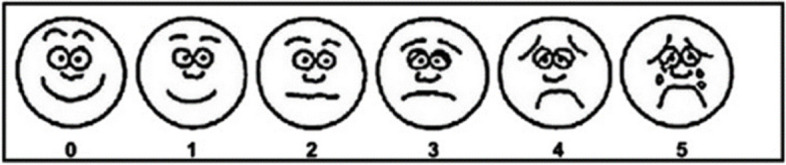
Visual analog scale. Comfort score. A score of 0 indicates no discomfort; 1, little bit discomfort; 2, little more discomfort; 3, even more discomfort; 4, whole lot more discomfort; 5, worst discomfort

The EEP, EIP, and comfort were measured with different nasal cannulas using the HFT-300 device. The flow rate was gradually increased from 0 to 60 L/min in increment of 10 L/min, and the recording began after the participant had taken five stable breaths. The respiratory pressure was recorded for 2 minutes, with a 5-minute rest before each flow rate adjustment (washout period). After measurement of each flow rate, comfort was assessed using a visual analogue scale (Fig. 2). This assessment involved asking the participants questions about airway-related symptoms, such as dryness of the mouth, nose, or throat; dysphagia; expiratory dyspnea; or throat pain. The procedure was repeated at intervals of more than 2 hours (washout period) using the other nasal cannulas until measurements had been obtained with all three nasal cannulas. The utilization of the three nasal cannulas was random. During the washout period, the nasal catheter and anesthesia catheter were removed to avoid discomfort to the subject due to prolonged wear.

### Assessment of differences in pressure and comfort using different devices

The EEP, EIP, actual flow in the pipeline and comfort were evaluated with the use of different devices. The participant underwent a trial with each of the two HFNC devices (the flow rate was set to 0, 10, 20, 30, 40, 50, and 60 L/min) and a 4-mm cannula. The pressure of each flow rate in the pipeline was recorded continuously for 2 minutes, with a 5-minute interval between different flow rates (elution period). After each flow rate was measured, a visual analogue rating scale was used to determine the score. The actual output flow was measured in the pipeline using a Thermal mass flowmeter (TSI 5300; TSI Incorporated, Shoreview, MN, USA) installed in the respiratory hose in front of the nasal catheter. The above process was repeated with the other device 24hous later (washout period). The order of using the two devices was random. The nasal catheters and anesthesia catheters were removed during the washout period.

### Statistical analysis

The data were analyzed using SPSS Version 25.0 (IBM Corp., Armonk, NY,USA). Data conforming to a normal distribution with as confirmed by the Shapiro–Wilk test are expressed as mean ± standard deviation. Data not conforming to a normal distribution are expressed as median and interquartile range. The Wilcoxon signed rank test was used to compare the EEP, EIP, and comfort level of different nasal catheters and devices, and the Mann Whitney U test was used to compare the differences in pressure between sexes. Differences in actual output flow rates were evaluated using paired t-tests. Multiple linear regression was used to determine predictors of EEP. The regression models were visually assessed using residual histograms and residual versus predicted plots. A *P*-value of < 0.05 was considered statistically significant. Our power analysis was conducted prior to the experiment. Considering a type 1 error of 0.05 and power of 90% we calculated an initial sample size of 30 participants. To allow for a 10% dropout rate, we aim for a sample size of at least 34 participants (Table S1).

## Results

### Basic information of participants

In total, 35 healthy volunteers were recruited in this study, including 16 men and 19 women. Their mean age was 24.54 ± 1.98 years, and their mean BMI was 21.05 ± 2.01 kg/m^2^ (Table 1).

**Table 1:** Basic information of research subjects

	Total	Male	Female
Age(years)	24.54 ± 1.98	24 ± 1.10	25 ± 2.43
height(cm)	167.37 ± 7.68	174 ± 3.54	161.79 ± 5.38
Body weight (kg)	59.23 ± 8.79	62.63 ± 12.46	54.26 ± 6.93
BMI (kg/m2)	21.05 ± 2.01	20.64 ± 3.84	20.70 ± 2.12
Total	35	16	19

### Differences in pressure and comfort between different nasal cannulas

**Fig. 3:**
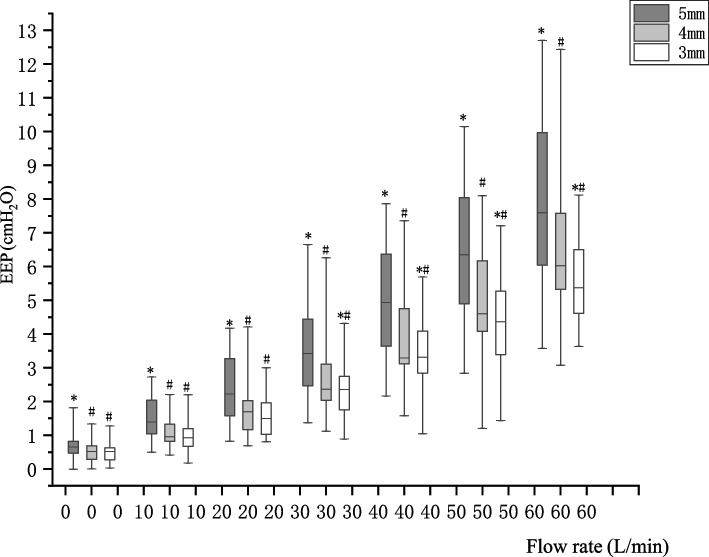
The EEP produced by different nasal cannulae in HFNC at different flow rates. # represents *P* < 0.05 compared to 5 mm cannula at the same flow rate, * represents *P* < 0.05 compared to 4 mm cannula

When the flow rate reached 30 L/min, the EEP produced by the three nasal cannulas began to differ (5 > 4 > 3 mm, *P* < 0.05), and the difference gradually became more obvious as the flow rate increased. When the flow rate reached 60 L/min, the EEP produced by the 5-mm tube was 7.18 (4.18) cmH_2_O, that produced by the 4-mm tube was 6.01 (2.21) cmH_2_O, and that produced by the 3-mm tube was 5.26 (1.88) cmH_2_O. The difference in EEP between the 4- and 5-mm tubes was more significant than that between the 3- and 4-mm tunes (Table 2, Fig. 3).

**Table 2:** EEP produced by different nasal cannulae in HFNC at different flow rates

Flowrate(L/min)	5 mm	4 mm	3 mm	5 mm–4 mm	4 mm–3 mm
0	0.65(0.32)*	0.51(0.40)^**#**^	0.47(0.35)^**#**^	0.14(0.45)	0.07(0.36)
10	1.34(0.98)*	0.94(0.46)^**#**^	0.86(0.55)^**#**^	0.50(1.03)	0.08(0.60)^**&**^
20	2.08(1.75)*	1.70(0.80)^**#**^	1.43(0.85)^**#**^	0.50(1.43)	0.13(0.58)^**&**^
30	3.12(2.06)*	2.31(0.82)^**#**^	2.31(0.92)^#^*	0.59(1.71)	0.23(0.90)^**&**^
40	4.92(2.53)*	3.25(1.19)^**#**^	3.25(1.13)^#^*	0.86(1.64)	0.11(0.98)^**&**^
50	6.21(2.93)*	4.47(1.81)^**#**^	4.24(1.82)^#^*	1.03(2.04)	0.41(1.35)^**&**^
60	7.18(4.18)*	6.01(2.21)^**#**^	5.26(1.88)^#^*	0.95(2.26)	0.07(0.36)

Values are median (IQR), **#** represents *P* < 0.05 compared to 5 mm cannula at the same flow rate, * represents *P* < 0.05 compared to 4 mm cannula, & represents *P* < 0.05 compared to EEP difference between 5 mm and 4 mm cannula at the same flow

**Fig. 4:**
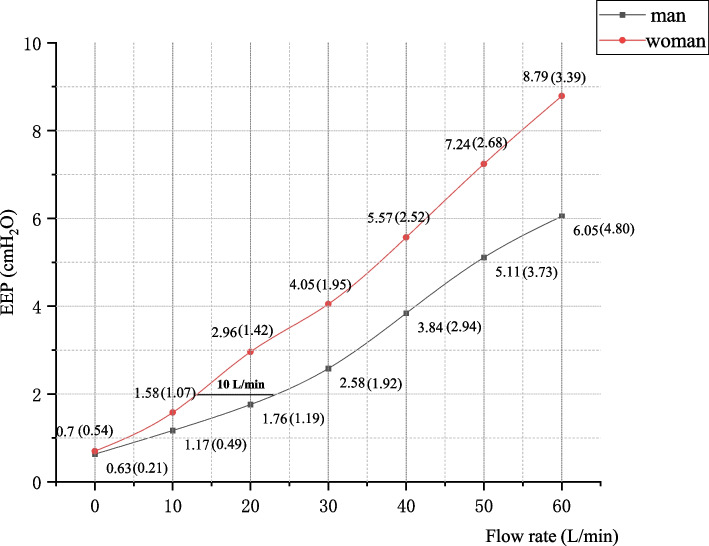
The EEP of 5 mm nasal cannula with flow rate for different gender. Values are median (IQR). When the target pressure reaches 2 cmH_2_O and above, the difference of the required flow rate between different genders can be up to 10 L/min

We also found a significant difference in EEP between the two sexes when using the 5-mm cannula. When the flow rate was 20 L/min, the EEP was 1.76 (1.19) cmH_2_O in men and 2.96 (1.42) cmH_2_O in women. When the flow rate reached 60 L/min, the EEP was 6.05 (4.80) cmH_2_O in men and 8.79 (3.39) cmH_2_O in women. (Table 3, Fig. 4). Notably, the effect of the nasal cannula on the EIP was not as significant as that on the EEP (Table 4).

**Table 3:** The EEP produced by different nasal cannulae for different gender

Flow rate	5 mm cannula		4 mm cannula		3 mm cannulae	
(L/min)	Man	Woman	Man	Woman	Man	Woman
0	0.63(0.21)	0.70(0.54)	0.48(0.52)	0.49(0.37)	0.43(0.43)	0.53(0.38)
10	1.17(0.49)	1.58(1.07)	0.96(0.55)	0.95(0.66)	1.00(0.74)	0.85(0.43)
20	1.76(1.19)	2.96(1.42)^**#**^	1.31(0.61)	1.75(0.90)	1.06(0.79)	1.65(0.70)
30	2.58(1.92)	4.05(1.95)^**#**^	2.11(0.66)	2.49(1.69)^**#**^	2.04(1.10)	2.47(0.95)^**#**^
40	3.84(2.94)	5.57(2.52)^**#**^	3.20(0.75)	3.67(2.44)	2.91(1.92)	3.61(0.78)^**#**^
50	5.11(3.73)	7.24(2.68)^**#**^	4.47(0.99)	4.99(2.68)	3.81(2.15)	4.75(1.45)
60	6.05(4.80)	8.79(3.39)^**#**^	5.63(1.69)	6.69(2.68)^**#**^	4.98(1.80)	5.82(1.89)

The value is the median (IQR), # represents the significant difference in EEP between different genders under the same flow rate and the same nasal cannula, *P* < 0.05

**Table 4:** EIP produced by different nasal cannulae in HFNC at different flow rates

Flow rate(L/min)		EIP	
	5 mm	4 mm	3 mm
0	−0.82(0.48)	−0.68(0.58)	−0.66(0.35)
10	−0.58 (0.47)	−0.61(0.58)	− 0.43(0.56)^#^
20	−0.07(0.56)	− 0.08(0.55)	− 0.13(0.55)
30	0.19(0.63)	0.28(0.54)	0.22(0.80)
40	1.02(1.21)	0.96(0.79)	0.94(1.21)^#^
50	1.77(1.51)*	1.49(1.24)^#^	1.62(1.51)
60	2.88(1.89)*	2.27(1.41)^#^	2.26(1.56)^#^

Values are median (IQR), ^#^ represents *P* < 0.05 compared to 5 mm cannula at the same flow rate, * represents *P* < 0.05 compared to 4 mm cannula

**Fig. 5:**
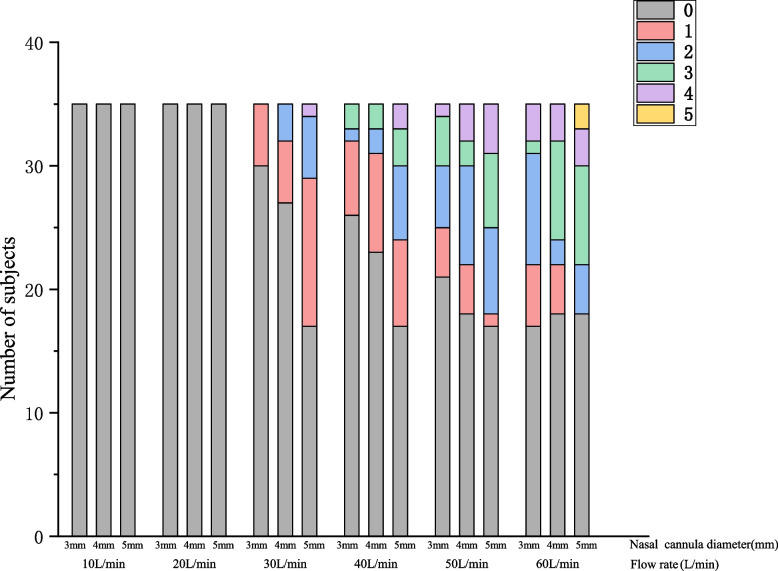
Dyspnea in different nasal cannulae

When the flow rate reached 30 L/min, the participants began to experience dyspnea, and the discomfort increased as the internal diameter of the nasal cannula increased (Fig. 5, Table S2). Other types of discomfort, (e.g., dryness of the mouth, nose, or throat dryness, dysphagia, and throat pain), showed no significant difference between the three nasal cannulas (Table S2).

### Linear regression of factors affecting EEP

An association was found between the EEP and the flow rate, sex, height and cannula size by linear regression. The EEP increased by 0.6 cmH_2_O per 1-mm increase in the cannula diameter, decreased by 0.3 cmH_2_O per 10-cm increase in participant height (with a 0.35 cmH_2_O decrease for men), and increased by 1 cmH_2_O when the flow rate increased by 10 L/min (R^2^ = 0.75, *P* < 0.05 for all variables in model) (Table 5).

**Table 5:** Linear regression

Predictors	Male gender	*P*-Value	95% confidence Interval
Height per 10 cm	−0.30	< 0.01	−0.5 −0.11
Flow per 10 L/min	1	< 0.001	0.97 1.06
Male gender	−0.35	< 0.05	−0.65 −0.05
Cannula diameter per 1 mm	0.60	< 0.001	0.49 0.71

### Effect of HFNC device model on nasal airway pressure and comfort

When the flow rate reached ≥20 L/min, and the EIP and EEP of the two devices were significantly different (H-80 M > HFT-300, *P* < 0.05), and the pressure difference as the flow rate increased. When the flow rate reached 60 L/min, the difference between the two devices reached 1.16 (1.09) cmH_2_O for EEP and 0.62 (1.30) cmH_2_O for EIP (Table 6). There was no significant difference in comfort between the two devices (Table S3).

**Table 6:** The pressure produced by different devices

Flow rate(L/min)	EEP			EIP		
	HFT-300	H-80 M	Gap of EEP	HFT-300	H-80 M	Gap of EIP
10	0.94(0.46)	1.07(0.57)	−0.03(0.02)	− 0.61(0.58)	−0.55(6.32)	− 0.01(0.31)
20	1.70(0.80)	1.93(0.85)*	0.20(0.55)	−0.08(0.55)	−0.06(0.54)^#^	0.11(0.53)
30	2.31(0.82)	2.87(1.34)*	0.37(0.59)	0.28(0.54)	0.64(0.67)^#^	0.17(0.47)
40	3.25(1.19)	4.39(1.88)*	0.66(1.01)	0.96(0.79)	1.54(1.07)^#^	0.48(0.78)
50	4.47(1.81)	5.71(22.78)*	1.07(1.22)	1.49(1.24)	2.25(1.30)^#^	0.72(0.74)
60	6.01(2.21)	7.40(3.21)*	1.16(1.09)	2.27(1.41)	3.12(1.45)^#^	0.62(1.30)

Values are median (IQR), * represents *P* < 0.05 compared to EEP of HFT-300 at the same flow rate, # represents *P* < 0.05 compared to EIP of HFT-300 at the same flow rate

**Fig. 6:**
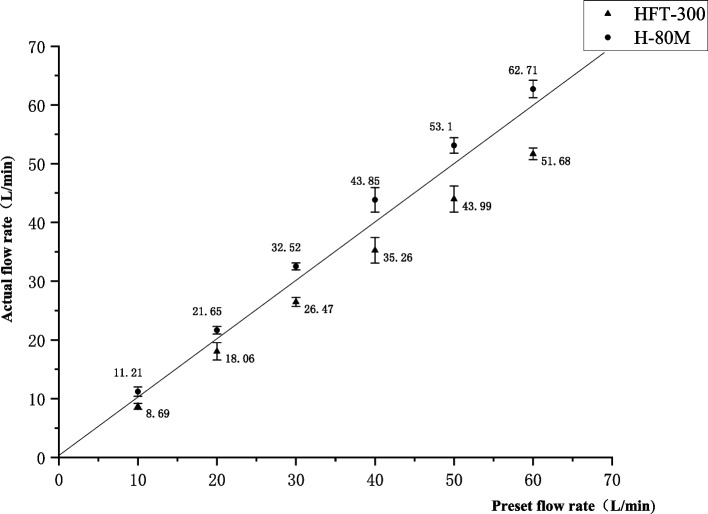
The actual output flow rate of different models of high-flow humidification therapy instruments. Values are median and standard deviation. The closer the data points are to the diagonal line, the closer the actual output flow is to the preset flow

Finally, the actual output flow rate was evaluated in the pipeline of the two devices. The flow rate measured by the H-80 M was significantly higher than that of the HFT-300 (*P* < 0.01) (Table 7, Fig. 6).

**Table 7:** The actual measured flow rate of devices

Pre-set flow rate(L/min)	HFT-300	H-80 M	Difference in actual output flow rate
10	8.69 ± 0.49	11.21 ± 0.78*	2.53 ± 0.85
20	18.06 ± 1.49	21.65 ± 0.65*	4.03 ± 1.24
30	26.47 ± 0.77	32.52 ± 0.59*	6.23 ± 0.51
40	35.26 ± 2.18	43.85 ± 2.08*	9.26 ± 1.85
50	43.99 ± 2.22	53.10 ± 1.31*	9.10 ± 2.43
60	51.68 ± 0.99	62.71 ± 1.49*	11.07 ± 1.84

Value is the mean ± SD, * represents *P* < 0.01 compared to HFT-300, output flow difference = output flow (H-80 M) - output flow (HFT-300)

## Discussion

In this study, the observed EEP in adults was different (either higher or lower) from that in earlier studies [14, 15]. This may have been due to the use of different geometries. Notably, the present study involved in vivo experiments, which are more closely related to clinical reality than previous airway models. Our study showed that height, nasal cannula size, sex, and flow rate can affect the EEP and that the EEP increases as size of the nasal cannula increases. Research by Hebbink et al. [10] found that whereas the pressure decreased with the cannula size in an adult model, the reverse was true for an infant models, this appears to contradict the findings of our research. Possible reasons for this discrepancy include the use of a larger nasal cannula, leading to a higher nostril occlusion rate (area of the prong divided by the nostril area), less gas leakage, and increasing EEP. However, a larger inner diameter can also result in a lower dynamic pressure of the HFNC jet, which reduces EEP. The occlusion rate in our experiment may be closer to that in the infant model in the above experiment (> 50%), although we did not measure the occlusion rate. The increase in cannula size mainly resulted in a decrease in gas leakage (increasing EEP) rather than a reduction in the HFNC jet (decreasing EEP).

Sex also has a significant effect on nasal airway pressure. The EEP was higher in women than in men in our study, and this difference between the two sexes was more significant with increasing nasal cannula size and flow. Groves et al. [2] also evaluated healthy volunteers and assessed the relationship of the airway pressure with body height and flow rate. They found that with each 10-cm increase in height, the EEP decreased by 0.5 cmH_2_O, and the mean EEP in men was 0.6 cmH_2_O lower than that in women. The pressure difference between the two sexes may be due to the smaller body size of women than men, resulting in a lower nasal volume and less air leakage at the same flow rate. Women may benefit more than men at the same flow rate, potentially providing a clinical reference for the adjustment of treatment parameters. When administering HFNC oxygen therapy to men, the flow rate should be appropriately increased to achieve the target treatment pressure and the desired therapeutic effect compared with women.

Race and age may also have an impact on the EEP, although these variables were not explored in our study. Corey et al. [16] evaluated the cross-sectional area of the nasal cavity in different ethnic groups and found a significant difference in the cross-sectional area between populations. In another study, the minimum cross-sectional area of the nasal cavity in neonates was approximately 0.114 cm^2^, which was much smaller than that of adults [17]. With the same nasal catheter size, a smaller nasal cross-sectional area is associated with more severe occlusion, less air leakage and a higher EEP level at the same flow rate. In our study, the effect of the nasal cannula diameter on the EIP was not as significant as that on the EEP. This may have been due to the fact that at the same flow, there is less leakage of air through the nose when inhaling than when exhaling. The diameter of the nasal catheter does not readily affect the EIP by affecting gas leakage. The effect of the nasal catheter on the EIP becomes apparent only when high flow exceeds the inspiratory flow rate, resulting in the significant gas leakage.

Although an increased inner diameter of the nasal cannula leads to increase the pressure in the nasal airway, it also results in more expiratory difficulties. Previous studies have shown that the most predominant discomfort associated with HFNC oxygen therapy in healthy adult volunteers in the awake state is dyspnea [18, 19], which is similar to our results. When the flow rate increases, the gas supplied by the ventilator causes some resistance to expiration. The body can protect the lungs from dry or cold inhaled air by reducing the airflow in the upper airways and trachea [20]. The warmed humidified gas provided by the HFNC avoids airway spasm and facilitates the discharge of secretions from the airway, improving comfort and tolerability [21, 22]. Another study [23] showed that the humidity of inhaled gas during HFNC oxygen therapy is influenced by the flow rate and inspiratory flow rate, but does not fall below 30 mg/L, remaining sufficient to meet the patients’ needs.

Chikata et al. [23] compared the humidification performance of different HFNC devices using a simulated lung system and found differences between the different machines. There were also differences between the data provided by the manufacturer and the clinical data obtained in the study [24]. At the same flow rate in our study, the actual output flow rates of the two devices measured in the pipeline were different, which may be the main reason for the different nasal airway pressures produced by the two machines. The differences in nasal airway pressures and output flow rates produced by the different devices in this experiment suggest that the working performance of the two machines differ. This should be of concern in the clinical setting. The absence of differences in the comfort levels between the two machines may have been due to the fact that the differences in performance between the two devices was not sufficient to cause changes in the subjective comfort level.

This study is limited by the fact that it was conducted on healthy volunteers rather than patients. Differences in comfort levels are likely to be present between patients and healthy volunteers. The comfort level may be the result of a combination of various physiological mechanisms [25]. However, expiratory pressures may not be markedly affected.

## Conclusion

The nasal cannula diameter, flow rate, sex, height, and device type can affect the nasal airway pressure, and the nasal cannula diameter and flow rate have an impact on comfort of HFNC oxygen therapy. Clinical should be aware of the impact of these factors on pressure and comfort.

### Supplementary Information

**Additional file 1.**

## Data Availability

The datasets used and/or analysed during the current study are available from the corresponding author on reasonable request.

## Abbreviations

HFNC: High-flow nasal cannula
EEP: End-expiratory pressure
EIP: End inspiratory pressure
SD: Standard deviation
IQR: Median and interquartile range

## References

[1] Basile MC, Mauri T, Spinelli E, Dalla Corte F, Montanari G, Marongiu I (2020). Nasal high flow higher than 60 L/min in patients with acute hypoxemic respiratory failure: a physiological study. Crit Care..

[2] Groves N, Tobin A (2007). High flow nasal oxygen generates positive airway pressure in adult volunteers. Aust Crit Care..

[3] Parke R, McGuinness S, Eccleston M (2009). Nasal high-flow therapy delivers low level positive airway pressure. Br J Anaesth..

[4] Guérin C, Cour M, Degivry F, Argaud L, Louis B (2022). A bench comparison of the effect of high-flow oxygen devices on work of breathing. Respir Care..

[5] Rittayamai N, Phuangchoei P, Tscheikuna J, Praphruetkit N, Brochard L (2019). Effects of high-flow nasal cannula and non-invasive ventilation on inspiratory effort in hypercapnic patients with chronic obstructive pulmonary disease: a preliminary study. Ann Intensive Care..

[6] Mauri T, Alban L, Turrini C, Cambiaghi B, Carlesso E, Taccone P (2017). Optimum support by high-flow nasal cannula in acute hypoxemic respiratory failure: effects of increasing flow rates. Intensive Care Med..

[7] García-Rivero JL, Esquinas C, Barrecheguren M, Bonnin-Vilaplana M, García-Sidro P, Herrejón A (2017). Risk factors of poor outcomes after admission for a COPD exacerbation: multivariate logistic predictive models. Copd..

[8] Renda T, Corrado A, Iskandar G, Pelaia G, Abdalla K, Navalesi P (2018). High-flow nasal oxygen therapy in intensive care and anaesthesia. Br J Anaesth..

[9] Sivieri EM, Gerdes JS, Abbasi S (2013). Effect of HFNC flow rate, cannula size, and nares diameter on generated airway pressures: an in vitro study. Pediatr Pulmonol..

[10] Hebbink RHJ, Duiverman ML, Wijkstra PJ, Hagmeijer R (2022). Upper airway pressure distribution during nasal high-flow therapy. Med Eng Phys..

[11] Xu Z, Li P, Zhang C, Ma D (2022). Effect of heated humidified high-flow nasal cannula (HFNC) oxygen therapy in dyspnea patients with advanced cancer, a randomized controlled clinical trial. Support Care Cancer..

[12] Tan D, Walline JH, Ling B, Xu Y, Sun J, Wang B (2020). High-flow nasal cannula oxygen therapy versus non-invasive ventilation for chronic obstructive pulmonary disease patients after extubation: a multicenter, randomized controlled trial. Crit Care..

[13] Yao JJ, Peng MM, Zou GH, He KX, Ma DD (2020). Effects of flow on carbon dioxide washout and nasal airway pressure in healthy adult volunteers during the constant-flow mode in a non-invasive ventilator. Chin Med J (Engl)..

[14] Pinkham M, Tatkov S (2020). Effect of flow and cannula size on generated pressure during nasal high flow. Crit Care..

[15] Adams CF, Geoghegan PH, Spence CJ, Jermy MC (2018). Modelling nasal high flow therapy effects on upper airway resistance and resistive work of breathing. Respir Physiol Neurobiol..

[16] Corey JP, Gungor A, Nelson R, Liu X, Fredberg J (1998). Normative standards for nasal cross-sectional areas by race as measured by acoustic rhinometry. Otolaryngol Head Neck Surg..

[17] Pedersen OF, Berkowitz R, Yamagiwa M, Hilberg O (1994). Nasal cavity dimensions in the newborn measured by acoustic reflections. Laryngoscope..

[18] Yao J, Li W, Peng M, He K, Ma D, Lu H (2022). The comfort assessment in healthy adults during constant-flow mode in noninvasive ventilator. Clin Respir J..

[19] Yu CC, Huang CY, Hua CC, Wu HP (2022). High-flow nasal cannula compared with continuous positive airway pressure in the treatment of obstructive sleep apnea. Sleep Breath.

[20] Fontanari P, Zattara-Hartmann MC, Burnet H, Jammes Y (1997). Nasal eupnoeic inhalation of cold, dry air increases airway resistance in asthmatic patients. Eur Respir J..

[21] Spoletini G, Alotaibi M, Blasi F, Hill NS (2015). Heated humidified high-flow nasal oxygen in adults: mechanisms of action and clinical implications. Chest..

[22] Boccatonda A, Groff P (2019). High-flow nasal cannula oxygenation utilization in respiratory failure. Eur J Intern Med..

[23] Chikata Y, Izawa M, Okuda N, Itagaki T, Nakataki E, Onodera M (2014). Humidification performance of two high-flow nasal cannula devices: a bench study. Respir Care..

[24] Thiéry G, Boyer A, Pigné E, Salah A, De Lassence A, Dreyfuss D (2003). Heat and moisture exchangers in mechanically ventilated intensive care unit patients: a plea for an independent assessment of their performance. Crit. Care Med..

[25] Mauri T, Galazzi A, Binda F, Masciopinto L, Corcione N, Carlesso E (2018). Impact of flow and temperature on patient comfort during respiratory support by high-flow nasal cannula. Crit Care..

