# Supplementary material for: Factors influencing nasal airway pressure and comfort in high-flow nasal cannula oxygen therapy: a volunteer study
Source: BMC Pulm Med. 2023 Nov 20;23:449. doi: 10.1186/s12890-023-02752-6 (PMC10658813; doi:10.1186/s12890-023-02752-6)
Supplement: Supplementary file 1 — Additional file 1. [file 12890_2023_2752_MOESM1_ESM.docx]

Table S1 Sample size at different flow rates in pre-experiment

| Flow rate (L/min) | 20 | 30 | 40 | 50 | 60 | 20 | 30 | 40 | 50 | 60 |
| --- | --- | --- | --- | --- | --- | --- | --- | --- | --- | --- |
| Gap of nasal cannulae pressure | 5mm-4mm | 5mm-4mm | 5mm-4mm | 5mm-4mm | 5mm-4mm | 4mm-3mm | 4mm-3mm | 4mm-3mm | 4mm-3mm | 4mm-3mm |
| Mean | 5.19 | 7.79 | 11.08 | 13.97 | 13.95 | 3.56 | 6.80 | 7.01 | 8.03 | 10.88 |
| SD | 8.35 | 7.28 | 9.55 | 11.58 | 15.41 | 5.70 | 8.13 | 8.36 | 8.10 | 12.30 |
| Sample Size | 30 | 12 | 10 | 10 | 15 | 29 | 18 | 17 | 13 | 16 |
| Expected Number of Dropouts | 4 | 2 | 2 | 2 | 2 | 4 | 2 | 2 | 2 | 2 |
| Dropout-Inflated Enrollment sample size | 34 | 14 | 12 | 12 | 17 | 33 | 20 | 19 | 15 | 18 |
| Dropout-Inflated Enrollment sample size= Sample Size/ (1- Dropout Rate), with “Dropout-Inflated Enrollment sample size” always rounded up. For example, 30/ (1-0.1) =33.333, count as 34 subjects. The expected number of dropouts= Dropout-Inflated Enrollment sample size- Sample Size | | | | | | | | | | |

| Flow (L/min) | | mouth, nose, or throat dryness | | | | | dysphagia | | | throat pain | | | expiratory dyspnea | | | |
| --- | --- | --- | --- | --- | --- | --- | --- | --- | --- | --- | --- | --- | --- | --- | --- | --- |
| diameter | 3mm | | 4mm | 5mm | | 3mm | 4mm | 5mm | 3mm | 4mm | | 5mm | 3mm | | 4mm | 5mm |
| 10 | 0 | | 0 | 0 | | 0±0 | 0±0 | 0±0 | 0±0 | | 0±0 | 0±0 | | 0 | 0 | 0 |
| 20 | 0 | | 0 | 0 | | 0±0 | 0±0 | 0±0 | 0±0 | | 0±0 | 0±0 | | 0 | 0 | 0 |
| 30 | 0.4±0.91 | | 0.26±0.61 | 0.14±0.43 | | 0±0 | 0±0 | 0±0 | 0±0 | | 0±0 | 0±0 | | 0.14±0.36 | 0.31±0.63 | 0.74±0.91*# |
| 40 | 0.46±0.95 | | 0.31±0.80 | 0.20±0.47 | | 0.11±0.40 | 0.23±0.60 | 0.14±0.50 | 0.11±0.40 | | 0.23±0.60 | 0.14±0.50 | | 0.4±0.81* | 0.51±0.85# | 1.03±1.25*# |
| 50 | 0.8±1.21 | | 0.37±0.69 | 0.49±1.07 | | 0.23±0.77 | 0.17±0.57 | 0.29±0.83 | 0.23±0.77 | | 0.17±0.57 | 0.29±0.83 | | 0.86±1.21* | 1.09±1.34# | 1.40±1.52*# |
| 60 | 1.17±1.34 | | 0.63±0.84 | 0.69±1.35 | 0.34±0.94 | | 0.37±0.94 | 0.37±0.94 | 0.34±0.94 | | 0.37±0.94 | 0.37±0.94 | | 1.09±1.29* | 1.26±1.50# | 1.54±1.74*# |
| # represents *P*<0.05 compared with 3mm nasal cannula at the same flow rate, * represents *P*<0.05 compared with 4mm nasal cannula at the same flow rate | | | | | | | | | | | | | | | | |

Table S2 Comfort of different nasal cannulae

| Flow（L/min） | expiratory dyspnea | | | mouth, nose, or throat dryness | | | | dysphagia | | | | throat pain | |
| --- | --- | --- | --- | --- | --- | --- | --- | --- | --- | --- | --- | --- | --- |
| Device | | HFT-300 | H-80M | | HFT-300 | H-80M | HFT-300 | | H-80M | HFT-300 | | | H-80M |
| 10 | | 0±0 | 0±0 | | 0±0 | 0±0 | 0±0 | | 0±0 | | 0±0 | | 0±0 |
| 20 | | 0±0 | 0±0 | | 0±0 | 0±0 | 0±0 | | 0±0 | | 0±0 | | 0±0 |
| 30 | | 0.31±0.63 | 0.4±0.65 | | 0.26±0.61 | 0.26±0.61 | 0±0 | | 0±0 | | 0±0 | | 0±0 |
| 40 | | 0.51±0.85 | 0.86±1.06 | | 0.31±0.80 | 0.31±0.72 | 0.23±0.60 | | 0.29±0.71 | | 0±0 | | 0±0 |
| 50 | | 1.09±1.34 | 1.03±1.29 | | 0.37±0.69 | 0.23±0.60 | 0.17±0.57 | | 0.09±0.37 | | 0±0 | | 0±0 |
| 60 | | 1.26±1.50 | 1.4±1.56 | | 0.63±0.84 | 0.49±0.82 | 0.37±0.94 | | 0.26±0.74 | | 0.26±0.56 | | 0.23±0.56 |

Table S3 Comfort of different HFNC devices
